# Genomic Analysis of a New Estrogen-Degrading Bacterial Strain, *Acinetobacter* sp. DSSKY-A-001

**DOI:** 10.1155/2019/2804134

**Published:** 2019-06-02

**Authors:** Qing Qiu, Ping Wang, Hui Kang, Yu Wang, Kejian Tian, Hongliang Huo

**Affiliations:** ^1^School of Life Sciences, Northeast Normal University, No. 5268, Renmin Main Street, Nanguan District, Changchun City, Jilin Province, China; ^2^School of Environment, Northeast Normal University, No. 2555 Jingyue Avenue, Changchun City, Jilin Province, China

## Abstract

In this study, we isolated a new estrogen-degrading bacterium from a soil sample collected near a pharmaceutical factory in Beijing, China. Morphological observations, physiological and biochemical analyses, and sequence analysis showed that the strain was in the genus *Acinetobacter*, and it was named DSSKY-A-001. The estrogen degradation rate and growth density of strain DSSKY-A-001 were determined by high-performance liquid chromatography and a growth assay using a microplate reader, respectively. The estrogen degradation rate was 76% on the third day and 90% on the sixth day of culture. Three kinds of estrogen metabolism intermediates were detected by high-performance liquid chromatography and mass spectrometry, and the estrogen metabolic pathway and possible estrogen-degrading enzymes were predicted. RT-PCR was used to verify whether the three putative enzymes, catechol 1,2-dioxygenase, dioxygenase, and 7*α*-hydroxysteroid dehydrogenase, were expressed in the strain. The results of the validation were consistent with the predictions that these three enzymes were present and expressed in *Acinetobacter* DSSKY-A-001. To further understand the estrogen-degrading activity of the strain at the genetic level, we sequenced the genome and performed a functional gene annotation. Through this gene sequence analysis, we identified genes predicted to encode the previously detected enzymes, catechol 1,2-dioxygenase, dioxygenase, and 7*α*-hydroxysteroid dehydrogenase, as well as six other enzymes that may be involved in estrogen degradation. Therefore, a total of nine enzymes related to estrogen degradation were found.

## 1. Introduction

Environmental endocrine-disrupting chemicals (EDCs) can enter the bodies of humans and animals and interfere with the normal metabolism of the endocrine system. EDCs have various effects on the growth, development, and reproductive function of humans and animals. [1]. The bulk of the available information regarding EDCs is on compounds possessing estrogen-like activity [2]. Estrogens themselves, such as estrone (E1), 17*β*-estradiol (E2), estriol (E3), and artificially synthesized 17*α*-ethinylestradiol (EE2), have extremely potent biological activity and, when excreted, cause environmental pollution [3, 4]. E2 is considered to be the strongest environmental estrogen [5, 6]. 17*β*-estradiol (E2) is a natural estrogen and a typical environmental endocrine-disrupting factor and is the strongest of many environmental hormones. It is ubiquitous in a variety of environments, especially in water. As environmental estrogen contamination has become an increasingly serious problem, methods for the biodegradation of estrogen are receiving increased attention as a major means for removing environmental estrogen [7]. So it is very important to find the bacteria that can effectively degrade estrogen.

*Acinetobacter* has extensive metabolic ability to degrade some pollutants in the environment; *Acinetobacter* is a genus of Gram-negative bacteria that is nonmotile, cannot ferment glucose, is strictly aerobic, and produces catalase. With the increasing problem of oil pollution, a growing number of reports on the degradation of petroleum by *Acinetobacter* species have been published. In 2014, Chen et al. isolated a bacterial strain, *Acinetobacter* sp. XM-02, which are capable of effectively degrading petroleum in petroleum-contaminated soil samples [8]. However, there are relatively few reports on the degradation of estrogen by *Acinetobacter* species. In one study, Pauwels et al. isolated six strains capable of degrading E2 and E1 in activated sludge from sewage treatment plants, and two of the six strains were identified as *Acinetobacter* [9]. Ke et al. isolated three strains of estrogen-degrading bacteria from artificial aquifers, and one of these strains was identified as *Acinetobacter* [10]. According to reports, Table 1 lists four estrogen-degrading strains of Acinetobacter and compares it with our Acinetobacter DSSKY-A-001. The comparison includes estrogen degradation mechanism, degree of degradation, initial concentration of estrogen, culture, culture time, degradation rate, similarity of accession No., 16S sequence, and query length. Here, we isolated an estrogen-degrading bacterium, which we identified as a species of *Acinetobacter*, and characterized the estrogen degradation pathway in this strain. Finally, we predicted the estrogen degradation pathway of Acinetobacter DSSKY-A-001 and predicted the enzymes involved in the degradation process. In order to conduct in-depth research from the genetic level, we have sequenced and analyzed the whole genome.

## 2. Materials and Methods

### 2.1. Isolation and Observation of Strains

**Figure 1:**
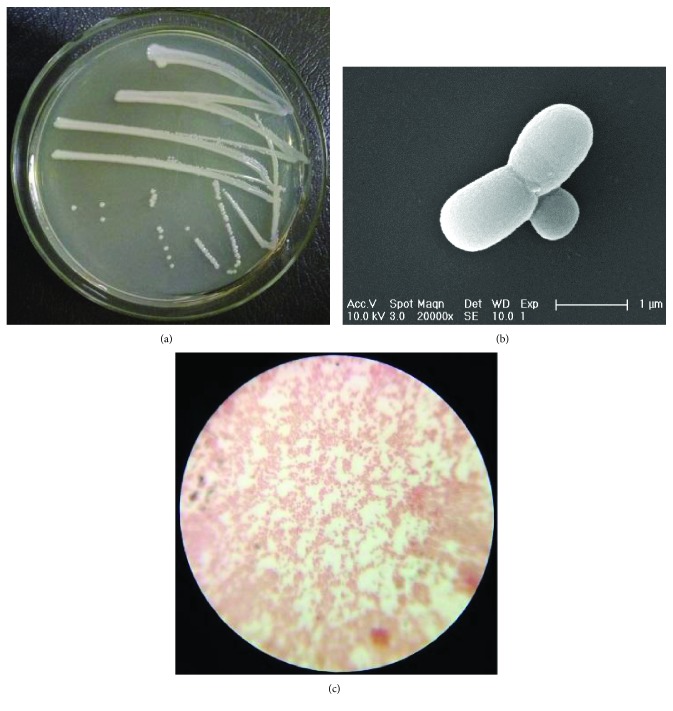
Morphological observations of the estrogen-degrading strain *Acinetobacter* DSSKY-A-001. (a) Colony morphology, (b) scanning electron micrograph, and (c) microscopic morphology; the magnification is 1,000 times.

Soil samples collected near a pharmaceutical factory in Beijing, China, were enriched and acclimated and then grown in medium containing estradiol (E2) as the sole carbon source. A method involving enrichment culture, dilution, and streaking was used to isolate a strain capable of degrading E2. The methods are as follows: one gram of soil sample is weighed and added to estradiol selection medium with a concentration of 5 mg/L. After 72 hours of culture in an oscillating incubator at 30°C and 120 rpm, 200 *μ*L is taken from the medium and inoculated into fresh estradiol selection medium of 10 mg/L. After 72 hours of culture in an oscillating incubator at 30°C and 120 rpm, the culture is continued in estradiol selection medium containing 20 mg/L, 40 mg/L, 60 mg/L, 80 mg/L, and 100 mg/L according to this method. Using the dilution coating plate method, dilute 1 mL of bacterial liquid to different concentrations of 10-2 to 10-8 with sterile water by a dilution coating plate method, coat the diluted bacterial liquid to LB solid culture medium with estradiol concentration of 100 mg/L; place the LB solid culture medium in a constant temperature incubator at 30°C; culture for 3 days; pick colonies with different shapes and colors; and perform multiple streaking on LB solid culture medium with estradiol concentration of 100 mg/L by a plate streaking method until a single colony is separated.  Figure 1(c) shows the scanning experiment procedure by an electron microscope: (1) sampling: first, add 2-3 mL of 2.5% glutaraldehyde fixative to the surface of the colony, and then, blow the colony with a gun. Pipette 100 *μ*L of the bacterial solution, add the bacterial solution to a 1 mL small centrifuge tube, centrifuge at 8000 rpm for 1 min, and discard the supernatant; (2) fixation: then, add 1 mL of 2.5% glutaraldehyde solution, mix with a vortex mixer, make the sample into a suspension, and place it in a refrigerator at 4°C for 18-24 hours; (3) washing: centrifuge at 8000 rpm for 3 min, discard the supernatant, resuspend for 5 min with deionized water, and repeat this step 3 times; (4) dehydration: perform gradient dehydration of the sample with 10%, 30%, 50%, 70%, 90%, 100%, and 100% (2 times total) of alcohol, each time 20 min, to ensure that the dehydration is clean (10% and centrifuge at 30% for 1 min and centrifuge at a concentration of 3 min later); (5) add 100-200 *μ*L of ethanol to resuspend. Pipette 10-50 *μ*L of the hanging droplets onto the silicon wafer stuck in the Petri dish, and then, cover the Petri dish cover with natural air drying; and (6) spray gold and observation: this step is done in the electron microscope room.

### 2.2. Experimental Reagents

In these experiments, we used 17*β*-estradiol (E2) (purity 98%, Shanghai Jingjing Biochemical Technology Co. Ltd.), other chemicals (analytical grade; Beijing Chemical Factory), a bacterial total RNA extraction kit (Bacterial RNA Kit; Haoran Biotech), the PrimeScript™ RT-PCR Kit (Dalian Bao Bioengineering Co.), the Bacterial Genomic DNA Extraction Kit (Tiangen Biochemical Technology Co. Ltd.), and an Enterobacteriaceae bacterial biochemical routine identification box (Guangdong Central Kai Microbial Technology Co. Ltd.). The following were also used in this study: inorganic salt medium, trace elements, LB medium, phosphate buffer stock solution (pH 7.0), phosphate buffer working solution (pH 7.0), estradiol mother liquor, and estradiol selection medium. The formulation of the estradiol mother liquor was obtained by weighing 0.5 g of estradiol, dissolved in methanol, and dilute to 50 mL to prepare a mother liquor having an estradiol concentration of 10 g/L.

The composition of the estradiol selection medium is as follows: 10 g/L estradiol mother liquor was added to 100 mL of inorganic salt medium to prepare final concentrations of estradiol of 5 mg/L, 10 mg/L, 20 mg/L, 40 mg/L, 50 mg/L, 60 mg/L, 80 mg/L, and 100 mg/L. The medium was placed in a constant temperature water bath, and a water bath at 60°C for 30min. After the methanol was completely evaporated, it was used.

### 2.3. Determination of Estrogen Concentration

Preparation of an estradiol standard curve: estradiol was prepared at concentrations of 10 mg/L, 20 mg/L, 40 mg/L, 60 mg/L, and 80 mg/L and analyzed by high-performance liquid chromatography (HPLC) to prepare a standard curve. A bacterial suspension with an OD at 600 nm (OD_600_) of 1.0 was added at 3% (*v*/*v*) to estradiol selection medium (with 50 mg/L estradiol), cultured at 30°C and 120 rpm for 6 days, and sampled every 24 hours. After pretreatment, the residual concentration of estradiol in the medium was determined by HPLC, and the cell density was measured as the OD_600_ using a microplate reader. The HPLC was conducted under the following conditions: UV detector, Dual*λ* Absorbance Detector, Water 2487; column, Zorbax Eclipse Plus C18 (150 × 4.6 mm, 3.5 mm); mobile phase, acetonitrile: water (1 : 1, *v*/*v*); detection wavelength, 275 nm; and flow rate, 0.8 mL/min, and amount injected, 10 *μ*L.

### 2.4. Determination of Estrogen Metabolites

The metabolites of E2 were determined by LC-MS using an API2000 mass spectrometer. The mass spectrometry conditions were as follows: EI source, negative ion scanning; steam temperature, 400°C; and capillary temperature, 200°C. Noninoculated samples were used as blank controls. A 10 *μ*L sample was injected into the LC-MS instrument for detection. The NIST mass spectrometry library was used to infer the metabolites of E2, and the metabolic pathway of E2 was further examined.

### 2.5. Detection of Estrogen-Degrading Enzymes by PCR and RT-PCR

To verify the presence and expression of dehydrogenase and dioxygenase in the *Acinetobacter* strain, we performed PCR and RT-PCR, respectively. The amplification was carried out using reagents from Beijing Quanjin Biotechnology Co. Ltd., including EasyTaq® DNA Polymerase (Cat. No. AP111-03) and High Pure dNTPs (Cat. No. AD101-02). The primers used are shown in Table 2. The isolated genomic DNA of the strain growing in the presence of E2 was used as a template for PCR. The PCR amplification conditions were as follows: 94°C for 5 min, followed by 35 cycles of 94°C for 30 sec, 55°C for 30 sec, and 72°C for 90 sec, and a final step at 72°C for 10 min. The obtained PCR product was subjected to agarose gel electrophoresis and recovered from the gel using the Gel Extraction Kit (Cat. No. D2500-02; Omega Bio-tek Inc., USA). Finally, sequencing was performed using corresponding amplification primers. Strain DSSKY-A-001 was inoculated into an inorganic salt medium containing E2 (50 mg/L) as the sole carbon source and incubated at 30°C and 120 rpm. After culturing for 3 days, total RNA was extracted using a bacterial total RNA extraction kit; the extracted RNA was then converted to cDNA by using an RT-PCR kit, and the obtained cDNA was used as a template for PCR. The PCR mixture is shown in Table 3. The PCR product was subjected to analysis by agarose gel electrophoresis, and the amplified fragment was detected. The fragments were recovered from the gel and sequenced. The primers used were designed using the primer design software Primer 5, and the sequences are shown in Table 2.

### 2.6. Genome Sequencing and Gene Annotation

Strain DSSKY-A-001 was inoculated in LB liquid medium and cultured at 30°C and 120 rpm for 24 h and then centrifuged at 4000 rpm for 10 min. The supernatant was discarded, and the cells were resuspended in 0.2 M phosphate buffer (pH 7.0). The cells were centrifuged again, and this process was repeated three times to wash the cells. Genomic DNA was extracted by using a bacterial genomic DNA extraction kit. The DNA concentration was determined using an ultratrace UV spectrophotometer (NanoDrop ND-1000). The DNA sample of the Rhodococcus strain DSSKP-R-001, which was tested by electrophoresis, was cut by a Covaris gTube into fragments of the desired size for construction of the library. After DNA damage repair and terminal repair, a hairpin-type linker was ligated using DNA ligase purification of the DNA fragments using AMPure PB beads at both ends of the DNA fragment to construct the SMRTbell library. The constructed library was quantified by Qubit, and the size of the inserted fragment was detected with Agilent 2100, followed by sequencing using the PacBio RSII platform. Whole-genome sequencing was performed using single-molecule sequencing. The raw data obtained by sequencing was filtered to obtain valid data. Starting from the clean data with various quality controls, SMRT portal software was used to perform genome assembly on the reads to obtain the initial assembly results. The reads were aligned to the assembly sequence, and the distribution of the sequencing depth was calculated. Based on the sequence length and alignment method, we discriminated whether the initial assembled sequence is chromosomal or a plasmid sequence and tested whether the sequence is looped.

We used GeneMarkS software and IslandPath-DIOMB software to predict gene islands, rRNAmmer software to predict rRNAs, tRNAscan software to predict tRNAs and tRNA secondary structure, Rfam software to predict sRNA, RepeatMasker software to predict scattered repeat sequences, and TRF software to predict tandem repeats. The predicted amino acid sequences of strain DSSKY-A-001 were compared with those in the COG, GO, KEGG, NR, and Swiss-Prot databases using NCBI BLAST. The genes of strain DSSKY-A-001 and their corresponding functions were combined to yield the annotation. For the assembled genomic sequence of the sequenced samples, combined with the predicted results of the coding genes, the Circos software [11] was used to display the sample genome, and the noncoding RNA and gene function annotations were analyzed to construct a genome-wide map of the strain DSSKY-A-001.

### 2.7. Accession Numbers

The genomic sequence of *Acinetobacter* DSSKY-A-001 has been deposited in GenBank under accession number CP027365.

## 3. Results and Discussion

### 3.1. Strain Identification and Preservation

#### 3.1.1. Morphological Observations

The growth status of the strain on LB agar medium is shown in Figure 1(a). The colonies were round, slightly convex, smooth and opaque in appearance, complete in edge, easy to pick, and milky white. Gram staining, which is shown in Figure 1(b), was negative. Electron microscopy (Figure 1(c)) showed rod-shaped bacteria with no flagella.

#### 3.1.2. Identification of Strain by 16S rDNA Sequencing

**Figure 2:**
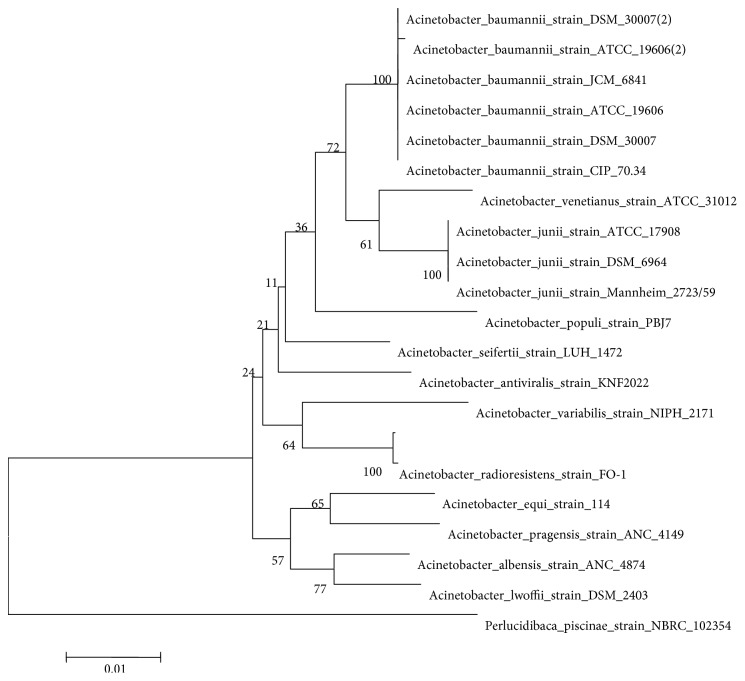
Evolutionary tree based on the 16S rDNA sequence. The phylogenetic dendrogram showed the relationship between the 16S rRNA sequence and DSSKY-A-001 and other closely related Acinetobacter strains, and the 16S rRNA sequence of Perlucidibaca_piscinae_strain_NBRC_102354 was used as the explant. Calculation of the phylogenetic dendrogram was based on neighbor-joining analysis with bootstrapping. Bootstrap confidence values were based on 1,000 iterations. The bar indicates a genetic distance of 0.01.

The 16S rDNA sequence of the strain was amplified by PCR using the universal primers shown in Table 4, and the components of the PCR mixture are shown in Table 5. The PCR product was sequenced, the sequencing results were aligned with the 16S rDNA sequences of reference strains in the GenBank database, and the phylogenetic position of the strain was determined based on the 16S rDNA sequence alignment. The 16S rDNA sequences of 19 strains of *Acinetobacter* were selected and compared with the sequence of the new strain as well as Acinetobacter_radioresistens_strain_FO-1, and a phylogenetic tree was generated by the neighbor-joining method using MEGA 5.2.1 software (Figure 2). The results showed that the homology between the new strain and *Acinetobacter_radioresistens_strain_FO-1* was 99%. The 16S sequence ID number of the *Acinetobacter_radioresistens_strain_FO-1* strain is NR_026210.1; therefore, the strain was initially identified as *Acinetobacter* and was named DSSKY-A-001. Strain DSSKY-A-001 was deposited in the General Microbiology Center (CGMCC) of the China Microbial Culture Collection Management Committee under accession number 12393. The 16S sequence ID number of the Acinetobacter DSSKY-A-001 is CP027365.1 in NCBI.

### 3.2. The Relationship between Estrogen Degradation and the Growth of Strain DSSKY-A-001

**Figure 3:**
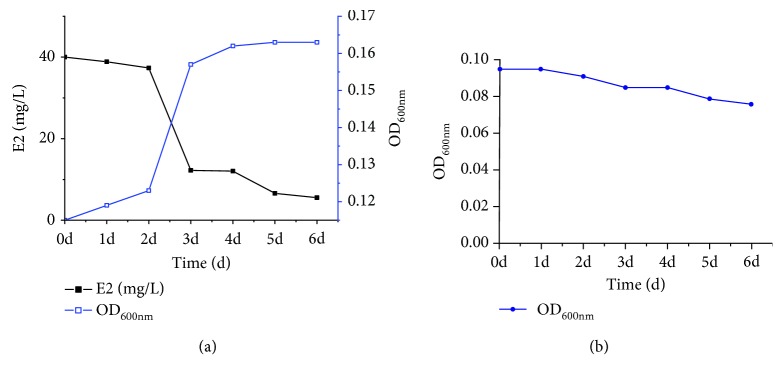
Growth and estrogen degradation by strain DSSKY-A-001: (a) experimental group (plus E2); (b) experimental group (plus E2).

The estradiol degradation rate and growth density of strain DSSKY-A-001 were determined by HPLC and a growth assay using a microplate reader, respectively. As shown in Figure 3(a), strain DSSKY-A-001 was able to grow on medium containing estradiol as the sole carbon source, and the degradation rate was 90% when cultured to for six days.  Figure 3(b) is a blank control without adding E2 plus strain. Through Table 1, we found that our strains have a relatively rapid degradation effect on high concentrations of estrogen.

### 3.3. Analysis of Estrogen Metabolites

**Figure 4:**
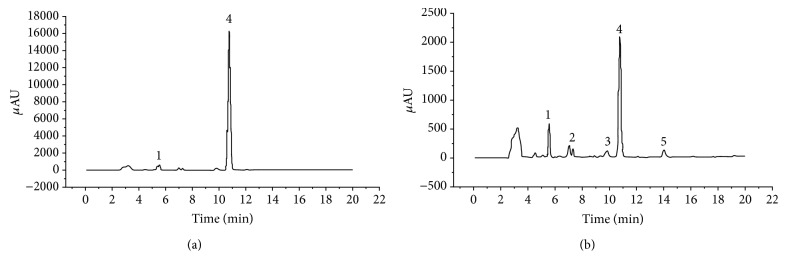
High-performance liquid chromatography (HPLC) of the intermediate products of E2 degradation by strain DSSKY-A-001. (a) HPLC of a blank sample (HPLC of samples with only E2 but no strain) and (b) HPLC of the culture at 6 d.

The metabolites produced during the degradation of E2 by strain DSSKY-A-001 were determined by HPLC and mass spectrometry (Figure 4).

The metabolites produced during E2 degradation by strain DSSKY-A-001 were detected by HPLC. In Figure 4(a), an E2 peak (peak 4) is present at a retention time of 10.75 min. Peak 1 is a background substrate peak. Peak 5 was present in the strain DSSKY-A-001 culture for the first 1–4 d, and peaks 2, 3, and 5 were present on days 5 and 6 (Figure 4(b)); Figure 4(b) shows a high-performance liquid chromatogram from the sixth day of culture. The results showed that when strain DSSKY-A-001 was cultured for 6 d, peaks appeared at retention times of 7, 10, and 14 min, suggesting the presence of three metabolites.

**Figure 5:**
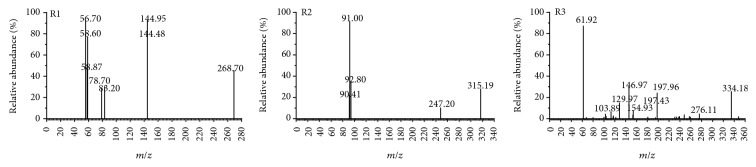
Mass spectra of the intermediate products of E2 degradation by strain DSSKY-A-001. In the mass spectrum, the abscissa indicates the mass-to-charge ratio (*m*/*z*) value of the ion, and the value of the mass-to-charge ratio from left to right increases. The ordinate indicates the intensity of the ion current, usually expressed as relative intensity; i.e., the strongest ion current intensity is set to 100%, and the ratio of other ion peak intensities to it is relative abundance.

Supplementary figure S1 shows the results of a metabolite scan using a first-order mass spectrometer. According to the primary mass spectrometry analysis of the intermediate metabolites and related research reports [9, 12], the E2 metabolic intermediates were preliminarily identified. The results showed that when strain DSSKY-A-001 was continuously cultured for 6 days, there were three E2 degradation products with mass-to-charge ratios of ~269, 315, and 334. Through mass spectrometry analysis, we determined that the charge-to-mass ratios (*m*/*z*) of the E2 metabolites might be 268.97, 315.02, and 334.11. The fragment ions of the three metabolites were detected by two-stage mass spectrometry, and the detection results are shown in Figure 5. These three fragment ions are complete ions, indicating that all three substances are intermediate metabolites of E2. The three substances were searched against related literature and the NIST mass spectrometry library to determine their name and structure.

The characteristic peak of metabolite R1 appeared at 268.70 *m*/*z* and was determined to be estrone (E1). These results were consistent with those of Yu et al. in 2007 [13], as they found that E1 was an intermediate metabolite of E2. R1 in Figure 5 shows the secondary mass spectrum of E1. The characteristic peak of metabolite R2 appears at 315.19 *m*/*z*. Based on the intermediate products of E2 examined by Kurisu et al., we presumed that this peak was 4-(3a-methyl-3,7-dodecane-6H-cyclopentadiene[a]naphthalene-6-Subunit)-2-methoxy-3-butenoic acid [14]. The characteristic peak of metabolite R3 appeared at 334.18 *m*/*z*, and based on the E2 metabolic intermediate reported by Yu et al., we identified this as (Z)-8-(7a-methyl-1n 5-dioxo-octahydro-1H-inden-4-yl)-2o-6-dioxy-4-butenoic acid [15].

### 3.4. Prediction of the Estrogen Metabolic Pathway

**Figure 6:**
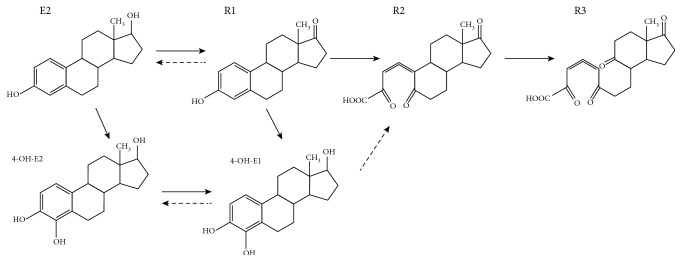
Proposed E2 degradation pathway in strain DSSKY-A-001.

Based on our analysis of the estrogen metabolites, we predicted the estrogen metabolic pathway in strain DSSKY-A-001, which is shown in Figure 6. Degradation of E2 usually begins with its conversion to E1 [12, 16], and E2 is converted to R1. In strain DSSKY-A-001, the hydroxyl group at the C-17*β* position of E2 is oxidized to a ketone group; that is, one molecule of hydrogen is removed from E2 to form E1, which is then further degraded to other products [17], which may be dehydrogenated. It has been reported that E1 is further degraded to R2 [14]; in this step, strain DSSKY-A-001 cleaves ring A of E1 to form R2, which may involve oxygenase. To further degrade R2 to R3, strain DSSKY-A-001 cleaves ring B of R2 to form R3, which may involve oxygenase.

E1 may also be further degraded to 4-OH-E1. In 1966, Coombe et al. first proposed that E1 degradation is catalyzed by dioxygenase at the A-ring, converting E1 to 4-OH-E1 [18]. It has been reported that 4-OH-E2 and 4-OH-E1 can be interconverted and that both can be further degraded by elemental cleavage. 4-OH-E1 can be converted to R2 by adding one molecule of oxygen [17]. Since 4-OH-E2 and 4-OH-E1 were not detected in this study, it is possible that E1 plus two molecules of oxygen are directly converted to R2, and this step may be catalyzed by oxygenase. R2 is unstable and may be converted to R3 by opening the B ring and adding one molecule of oxygen, which may also be catalyzed by oxygenase. The degradation pathway of estrogen is conjectured in our laboratory. The accuracy and comprehensiveness of estrogen degradation need to be further explored.

### 3.5. Estrogen Degradation-Related Enzymes

#### 3.5.1. Estrogen Dehydrogenase

The estrogen-degrading dehydrogenase in strain DSSKY-A-001 belongs to the short-chain dehydrogenase/reductase (SDR) family of enzymes. SDR enzymes are widely present in organisms (bacteria, archaea, and eukaryotes) and are capable of metabolizing a range of substrates, including aliphatic aldehydes and ketones, monosaccharides, steroid hormones, prostaglandins, flavonoids, polycyclic aromatic hydrocarbons, and retinoic acid, among others. Many of the substrates of SDRs are important signaling molecules that function within eukaryotic cells or between cells [18, 19].

*(1) 7α-Hydroxysteroid dehydrogenase*. 7*α*-Hydroxysteroid dehydrogenase (7*α*-HSD) is found in many bacteria and the livers of mammals, and it catalyzes the dehydrogenation of hydroxyl groups at the C-7 position of the steroidal skeleton of bile acids [20, 21]. The three-dimensional structures of two 7*α*-HSDs from *Escherichia coli* [21] and *Brucella melitensis* [22] have been determined, which indicate that they belong to the SDR family, and exist as dimers or tetramers. We speculate that 7*α*-hydroxysteroid dehydrogenase may catalyze the removal of one molecule of hydrogen from E2 to generate E1.

### 3.6. Estrogen-Degrading Dioxygenase

Bacteria have two key enzymes that are involved in the metabolism of polycyclic aromatic hydrocarbons. One is dioxygenase, which initiates the reaction by catalyzing the addition of molecular oxygen to the benzene ring [23]; the other is catechol dioxygenase, which is the key enzyme that opens the aromatic ring. With the completion of open-loop cleavage, an intermediate product of the tricarboxylic acid cycle (TCA) is formed, which then enters the TCA cycle and undergoes complete oxidative decomposition [24].

#### 3.6.1. Dioxygenase

Dioxygenase is one of the key enzymes in the biodegradation of aromatic compounds and their derivatives. This enzyme can utilize a variety of substrates and is important for the degradation of many aromatic pollutants [25, 26].

#### 3.6.2. Catechol Dioxygenase

Catechol dioxygenase is a key enzyme for the biodegradation of aromatic compounds [27]. Catechol dioxygenases are mainly classified as catechol 1,2-dioxygenase (C12O) or catechol 2,3-dioxygenase (C23O), and both C12O and C23O are capable of opening a benzene ring to form catechol [28]. C12O was discovered by Harayama and Rekik [29].

### 3.7. Expression of Estrogen-Degrading Enzymes

**Figure 7:**
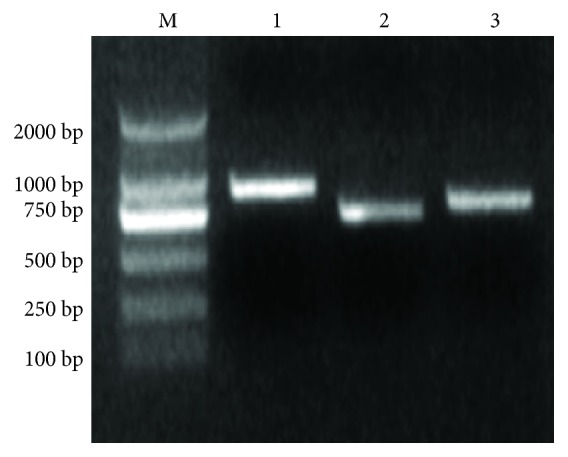
Agarose gel electrophoresis of PCR assay to detect estrogen-degrading enzyme genes. M: marker; lanes 1–3: PCR amplification products of (1) catechol 1,2-dioxygenase, (2) dioxygenase, and (3) 7*α*-hydroxysteroid dehydrogenase.

**Figure 8:**
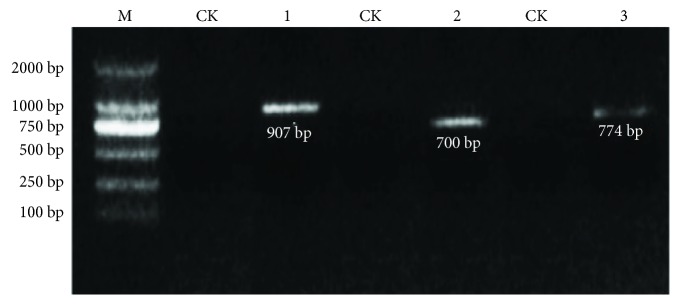
Agarose gel electrophoresis of RT-PCR assay to detect the expression of estrogen-degrading enzymes. M: marker; lanes 1–3: RT-PCR amplification products of (1) catechol 1,2-dioxygenase, (2) dioxygenase, and (3) 7*α*-hydroxysteroid dehydrogenase. CK: control group.

To verify the presence and expression of catechol 1,2-dioxygenase, dioxygenase, and 7*α*-hydroxysteroid dehydrogenase in our strain, we performed PCR and RT-PCR. The PCR amplification results for the corresponding target fragments are shown in Figure 7. The results of the RT-PCR assay are shown in Figure 8. The results showed that when strain DSSKY-A-001 was cultured in medium containing E2 as the sole carbon source, hydroquinone 1,2-dioxygenase, dioxygenase, and 7*α*-hydroxysteroid dehydrogenase mRNA are expressed.

### 3.8. Analysis of the Whole-Genome Sequence of Strain DSSKY-A-001

**Figure 9:**
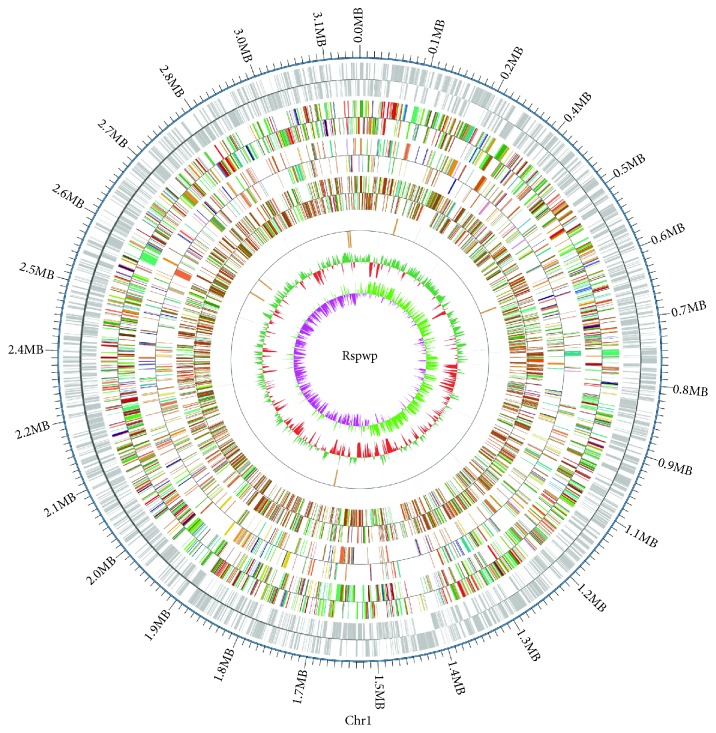
Whole-genome map of strain DSSKY-A-001; the genome of the strain DSSKY-A-001 is composed of a cycled chromosome with a length of 3132860 bp. The outermost circle is the position coordinates of the genomic sequence, from the outside to the inside, respectively, the coding gene, gene function annotation results (according to the actual project situation, may include COG, KOG, eggNOG, KEGG, and GO database annotation result information), ncRNA, genome GC content, and genomic GC skew value distribution.

To further investigate the molecular mechanism of estrogen degradation in strain DSSKY-A-001, we performed whole-genome sequencing.  Figure 9 shows the results of the whole-genome sequencing and assembly. The genome is not large but contains a very rich functional area, the proportion of 85.99% of the genome size. In addition to the gene-coding regions, more noncoding regions have functions of transcriptional regulation, posttranscriptional regulation, translational regulation, and epigenetic regulation. Some functional regions are also related to the diversity of species evolution. As shown in Table 6, the genome of strain DSSKY-A-001 consists of a single circularized chromosome. The outermost circle of the genome map shows the coordinates of the genomic sequence, and the different colors have different information. The rings from the outside to the inside indicate the following: the coding genes and gene function annotation results (by COG, KEGG, and GO results), ncRNAs, genomic GC content, and genomic GC skew distribution. We analyzed the number of genes associated with each COG category, which is shown in Table 7.

### 3.9. Analysis of Estrogen-Degrading Enzyme Genes

Through the functional gene annotation, we identified six enzymes related to estrogen degradation, three of which were analyzed by RT-PCR before sequencing, and the annotations in the sequencing results were also noted.

**Figure 10:**
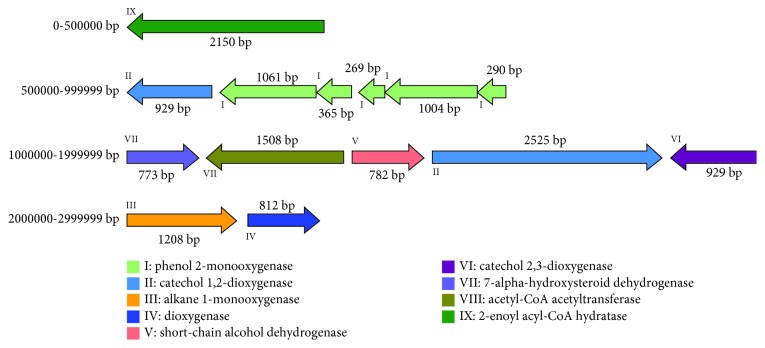
Location and organization of the identified estradiol-degrading enzymes in *Acinetobacter* sp. DSSKY-A-001.

After genome sequencing, we performed a BLAST analysis to compare the predicted amino acid sequences of *Acinetobacter* sp. DSSKY-A-001 to the gene annotations obtained from the COG, GO, KEGG, NR, and Swiss-Prot databases. The analysis predicted nine estrogen-degrading enzymes in *Acinetobacter* sp. DSSKY-A-001, which are shown in Table 8. The size and distribution of these nine estrogen-degrading enzymes in the strain are shown in Figure 10.

## 4. Conclusions

In this paper, a new estrogen (17*β*-estradiol)-degrading bacterial strain was isolated, and the strain in GenBank showing the highest 16S rDNA sequence homology with the isolate, strain DSSKY-A-001, was Acinetobacter_radioresistens_strain_FO-1 (99%). This suggested that DSSKY-A-001 belongs to *Acinetobacter*. Strain DSSKY-A-001 was cultured on medium containing estradiol as the sole carbon source, and the growth density and estradiol degradation rate of strain DSSKY-A-001 were determined. The results showed that with increased culture time, the density of the bacteria and the estradiol degradation rate gradually increased, and the degradation rate of E2 reached 90% on the sixth day of culture. The E2 metabolites produced by strain DSSKY-A-001 were determined by HPLC and MS, and three metabolites were identified. Based on this, a metabolic pathway of E2 degradation was proposed. In most E2 biodegradation pathways, whether aerobic, anoxic, or anaerobic, E2 is usually first dehydrogenated at the C-17 position to yield E1 [12, 16] and then further degraded to other products. However, Kurisu et al. found that during the degradation of E2 by estrogen-degrading bacteria isolated from soil, an intermediate metabolite of E2, 4-OH-E2, was present, indicating that E2 was hydroxylated at the C-4 position to yield 4-OH-E2 [14].

The degradation pathway of E1 was first reported by Coombe et al. in 1966, and in a study of E1 degradation by Nocardia sp. E110, it was first proposed that dioxygenase is involved in the cleavage of ring A in E1. Dioxygenase hydroxylates the C-4 position of ring A in E1 to convert it to 4-OH-E1 [30]. 4-OH-E2 can also be converted to 4-OH-E1 via dehydrogenation, and 4-OH-E2 and 4-OH-E1 can be further degraded to other substances via elemental cleavage or oxidatively decomposed via the tricarboxylic acid (TCA) cycle. In the proposed pathway, dehydrogenase is involved in the generation of E1, and oxygenase is involved in the conversion of E1 to R2 and R2 to R3. To determine the genes involved in the degradation of estrogen at the genetic level, we sequenced the genome of strain DSSKY-A-001, performed a functional gene annotation, and used other bioinformatic methods. Based on the annotation and analyses using the COG, KEGG, GO, SwissProt, and NR databases, nine estrogen degradation-related enzymes, including phenol hydroxylase, catechol 1,2-dioxygenase, alkane oxygenase, dioxygenase, short-chain dehydrogenase, catechol 2,3-dioxygenase, and 7*α*-hydroxysteroid dehydrogenase, were identified. Among the nine enzymes, we found the genes encoding the three enzymes we previously predicted, catechol 1,2-dioxygenase, dioxygenase, and 7-*α*-hydroxysteroid dehydrogenase. This report provides a theoretical basis for the in-depth study of the structure and function of estrogen-degrading enzymes through a bioinformatic analysis of these three enzymes.

## Figures and Tables

**Table 1:** Isolated estrogen-degrading bacteria.

Strain	Degradation ability and mechanism	Concentration	Degradation rate (%)	Time (d)	Accession No. (NCBI)	Similarity (%)	Query length	References
Acinetobacter sp. LHJ1 (Acinetobacter sp. AU783)	E2 to E1	E2: 500 *μ*g/L	100%	15 d	AY043368	96%	1462 bp	[10]
Acinetobacter sp. BP8	E1, E2, and E3	E2: 1.8 mg/L	100%	5 d	EF 198472	96%	1243 bp	[9]
Acinetobacter sp. BP10	E1, E2, and E3	E2: 5 mg/L	100%	2 d	EF 198473	97%	1236 bp	[9]
Acinetobacter DSSKY-A-001	E2 to E1	E2: 40 mg/L	90%	6 d	CP027365	100%	1404 bp	This study

**Table 2:** PCR primer sequences.

Degradation enzyme	Primer sequence (5′-3′)	Annealing temperature (°C)	Length of the genes
Catechol 1,2-dioxygenase	F: ATGAACCGTCAACAAATTGATGC	55	907 bp
	R: GGCGTCGTTCAACTTCGCTAG		
Dioxygenase	F: TGGTTCTCCCATGCTCGCTCT	55	700 bp
	R: ATTCCTAGAGCCCCCATAGAAAAAC		
7*α*-Hydroxysteroid dehydrogenase	F: ATGTATCAGTCCCTATTAGATTTATCC	55	774 bp
	R: TTAATCAAGGGTTTGTACTCCG		

**Table 3:** PCR mixture.

Component	Volume
Template DNA	1 *μ*L
Primer F (10 *μ*M)	0.5 *μ*L
Primer R (10 *μ*M)	0.5 *μ*L
10x Easy Taq Buffer	2.5 *μ*L
2.5 mM dNTPs	2 *μ*L
DNA polymerase (Taq polymerase)	0.5 *μ*L
ddH_2_O	18 *μ*L
Total volume	25 *μ*L

**Table 4:** Universal primers used for 16S rDNA amplification.

Primers	Primer sequences (5′-3′)
Upstream primer 27F	AGAGTTTGATCMTGGCTCAG
Downstream primer 1492R	TACGGYTACCTTGTTACGACTT

**Table 5:** PCR mixture for 16S rDNA amplification.

Components	Volume
Template DNA	1 *μ*L
Primer (F+R)	0.8 *μ*L
DNA polymerase (Taq polymerase)	10 *μ*L
ddH_2_O	8.2 *μ*L
Total volume	20 *μ*L

**Table 6:** Genome statistics.

Attribute	Value
Genome size (bp)	3,132,860
DNA G+C (bp)	41.61%
Gene coding	85.99%
Gene total size (bp)	2,693,802
Number of genes	2,963
tRNA	76
rRNA	21
5S rRNA	7
16S rRNA	7
23S rRNA	7
Genes assigned by COGs	2,174
Genes assigned by KEGG	1,770
Genes assigned by GO	2,036
Genes assigned by NR	2,877
Coding genes assigned by Swiss-Prot	1,347

**Table 7:** Number of genes associated with general COG functional categories.

Code	Number of genes	Description
A	1	RNA processing and modification
C	168	Energy production and conversion
D	30	Cell cycle control, cell division, and chromosome partitioning
E	202	Amino acid transport and metabolism
F	68	Nucleotide transport and metabolism
G	98	Carbohydrate transport and metabolism
H	136	Coenzyme transport and metabolism
I	185	Lipid transport and metabolism
J	209	Translation, ribosomal structure, and biogenesis
K	153	Transcription
L	93	Replication, recombination, and repair
M	142	Cell wall/membrane/envelope biogenesis
N	31	Cell motility
O	106	Posttranslational modification, protein turnover, and chaperones
P	145	Inorganic ion transport and metabolism
Q	74	Secondary metabolites biosynthesis, transport, and catabolism
R	226	General function prediction only
S	127	Function unknown
T	103	Signal transduction mechanisms
U	49	Intracellular trafficking, secretion, and vesicular transport
V	54	Defense mechanisms
W	20	Extracellular structures

**Table 8:** Estrogen degradation-related enzymes identified in *Acinetobacter* sp. DSSKY-A-001.

Gene ID	Enzyme	EC number
RspwpGM000877/000878/000880/000881/000882	Phenol hydroxylase	EC 1.14.13.7
RspwpGM000876/001688	Catechol 1,2-dioxygenase	EC 1.13.11.1
RspwpGM002178	Alkane oxygenase	EC 1.14.15.3
RspwpGM002188	Dioxygenase	EC 1.13.11
RspwpGM001672	Short chain dehydrogenase	EC 1.3.1.25
RspwpGM001812	Catechol 2,3-dioxygenase	EC 1.13.11.2
RspwpGM001333	7*α*-Hydroxysteroid dehydrogenase	EC 1.1.1.159
RspwpGM001515	Acetyl-CoA C-acetyltransferase	EC 2.3.1.9
RspwpGM000226/000580	Enoyl-CoA hydratase	EC 4.2.1.17

## Data Availability

The data used to support the findings of this study are included within the article.

## Supplementary Materials

Supplementary Materials Our article has done a lot of experiments, including primary and secondary mass spectrometry. Because of the limitation of the number of words and the length of the text, the first-order mass spectrum cannot be displayed in the text, and I feel that it is very helpful to the reader. Therefore, we put the relevant content of the first-level mass spectrometer into the supplementary materials and hereby explain the situation.
