# Supplementary material for: Genomic Analysis of a New Estrogen-Degrading Bacterial Strain, Acinetobacter sp. DSSKY-A-001
Source: Int J Genomics. 2019 Jun 2;2019:2804134. doi: 10.1155/2019/2804134 (PMC6589213; doi:10.1155/2019/2804134)
Supplement: Supplementary Materials — Our article has done a lot of experiments, including primary and secondary mass spectrometry. Because of the limitation of the number of words and the length of the text, the first-order mass spectrum cannot be displayed in the text, and I feel that it is very helpful to the reader. Therefore, we put the relevant content of the first-level mass spectrometer into the supplementary materials and hereby explain the situation. [file 2804134.f1.doc]

Figure S1: Mass spectra of the intermediate products of E2 degradation produced by strain DSSKY-A-001. (a–e) First order mass spectra of (a) blank sample only adds e2, no bacteria, (b) peak 2, (c) peak 3, (d) peak 4, and (e) peak 5.

Figure S1 shows the results of a metabolite scan using a first order mass spectrometer. The molecular weight of the compound is the result of deprotonation of the parent compound, i.e., [M-H]. Figure S1a shows a first-order mass spectrum of a blank sample, and Figure S1b–e are the first-order mass spectra of peaks 2, 3, 4, and peak 5, respectively. The E2 metabolic intermediates were preliminarily identified.

The mass to charge ratio of E2 is ~271. Comparison to the blank sample (Fig. S1a), showed that products with a mass-to-charge ratio of ~269 on days 1–6 of culture, and products having a mass-to-charge ratio of ~315 and 334 on days 5 and 6. The product with a mass-to-charge ratio of ~269 may be due to the hydrogen conversion of E2 by one molecule, and the generated substance may be estrone (E1). The amount of the product with a mass-to-charge ratio of ~269 gradually increased on the first 1–2 d, suggesting that it may be the conversion of E2 to E1. It gradually decreased on days 3–4, which may represent part of the E1 being converted to E2, and then on days 5–6, it gradually increased, which may be E2 or other products that are converted to E1. We speculated that the substance with a mass-to-charge ratio of 334.11 may also be a metabolite of E2. The materials with mass-to-charge ratios of 315.02 (Fig. S1b) and 334.11 (Fig. S1d) may also be metabolites of E2. The results showed that when strain DSSKY-A-001 was continuously cultured for 6 days, there were three E2 degradation products with mass-to-charge ratios of ~269, 315, and 334. Through mass spectrometry analysis, we determined that the charge-to-mass ratios (m/z) of the E2 metabolites might be 268.97, 315.02 and 334.11.
